# The impact of muscle mass, strength, and physical functioning on postural balance in older adults with sarcopenia

**DOI:** 10.1016/j.clinsp.2025.100784

**Published:** 2025-09-12

**Authors:** Cenyi Wang, Xinrong Jiao, Jiling Liang, Aming Lu

**Affiliations:** a School of Physical Education and Sports Science, Soochow University, Suzhou, China; b Faculty of Chinese Medicine Science, Guangxi University of Chinese Medicine, Nanning, China; c Department of Physical Education, Central South University, Changsha, China

**Keywords:** Aged, Postural balance, Sarcopenia

## Abstract

**Objectives:**

This study sought to investigate the effect of muscle mass, muscle strength, and physical function on postural balance in Sarcopenia.

**Methods:**

A total of 1026 subjects aged 60-years and older were screened for sarcopenia and assessed using clinical tests including the Romberg's test, Single Leg Standing Test (SLS), Five Times Sit to Stand Test (FTSST), Timed Up & Go Test (TUGT), and Y-Balance Test (YBT).

**Results:**

The authors identified distinct predictive patterns: 1) Hand grip strength(muscle strength) emerged as the dominant predictor of static balance performance, while 2) Dynamic balance outcomes were jointly influenced by gait speed (physical function), muscle strength, and age. Notably, dynamic balance demonstrated greater susceptibility to age-related skeletal muscle decline compared to static balance.

**Conclusion:**

These findings propose clinically actionable pathways: resistance training to target muscle strength deficits for static balance improvement, and combined strength-functional training protocols for dynamic balance rehabilitation in sarcopenia management.

## Highlights

• Revealed differential predictors of balance control in sarcopenia.
• Identified sex- and age-specific vulnerability patterns.
• Proposed targeted rehabilitation protocols.

## Introduction

Reduced physical activity and rising chronic disease rates in older adults contribute to functional impairments (e.g., frailty, cardiopulmonary decline), significantly compromising health-related quality of life and increasing healthcare system burdens.^1^ Age-related muscle atrophy is one of the most common types of muscle atrophy in humans.^2^ As sensory input decreases and slows with age, the reduction in physical activity and sensory function in older adults leads to a decline in their motor function and ability to live independently.^3^

Postural balance represents the body's ability to respond to visual, vestibular, and proprioceptive inputs to maintain stable posture through proper muscle control to prevent falls. Studies have shown that more than 60 % of falls in older adults are due to postural imbalances in the body. A variety of age-related changes in skeletal muscle function can affect the maintenance of postural balance and increase the risk of falls in older adults.^4^ Aging is associated with changes in the spindle of skeletal muscle fibers and their neural pathways, leading to a decrease in the sensitivity and the ability to integrate proprioceptive signals in older adults, thus altering their ability to control posture and ultimately decreasing the functional independence of the individual. Thus, good postural balance requires rapid synergies and interactions between various physiological and cognitive factors, including sufficient muscle strength, good sensation, appropriate nerve conduction, etc., to enable the body to respond quickly and accurately to sudden disturbances.^5^ Postural balance assessment in older adults involves two components (static and dynamic), which serve as crucial indicators of postural control capacity.^6^

Sarcopenia, an age-related syndrome characterized by progressive loss of muscle mass, strength, and physical function, ultimately impairs daily living activities. Sarcopenia is associated with elevated risks of falls, fractures, and physical disability in older populations, serving as a key contributor to age-related functional decline. Despite consensus from the sarcopenia experts on muscle-balance interactions, most evidence derives from Western populations using single-modality assessments, whereas Asian studies remain limited to small-sample biomechanical analyses. In recent years, as sarcopenia research has been gradually deepened by scholars in the fields of sports science and geriatrics, clinical experimental studies investigating the effects of functional independence and the incidence of falls in people with sarcopenia have attracted increasing attention.^7^^,^^8^ Previous studies have shown that patients with sarcopenia have significantly reduced muscle mass, muscle strength, and functional activity compared to normal older adults.^9^ Furthermore, some scholars have conducted studies on individuals with sarcopenia using force plates, foam balance tests, psychological anxiety scales, etc., and concluded that sarcopenia could significantly affect balance function.^10^^,^^11^ While sarcopenia exercise-nutrition interventions are well studied, critical gaps persist in understanding how skeletal muscle aging impairs postural balance and in developing targeted balance rehabilitation for affected older adults.^12^^,^^13^

Therefore, in this study, the common clinical balance function was used to explore the underlying relationship between diagnostic criteria and postural balance in sarcopenia, in order to provide a data reference for formulating exercise intervention programmes, improving postural balance in individuals with sarcopenia, and reducing the risk of falls. It is hypothesized that the static and dynamic balance function of the sarcopenia population would be significantly lower than that of the non-sarcopenia population, and that the static and dynamic balance performance of the sarcopenia population could be predicted and estimated by diagnostic indicators.

## Materials and methods

### Subjects

Subjects were recruited from February 2021 to December 2023 in communities in Suzhou, Jiangsu Province, for older adults aged 60-years or older. Recruitment methods included paper advertisements, WeChat, flyers, telephone, and on-site recruitment. The diagnosis of sarcopenia was based on the diagnostic criteria for sarcopenia proposed by the Asian Working Group for Sarcopenia (AWGS) in 2019.^14^ The diagnosis of sarcopenia consists of three components, namely muscle mass, muscle strength, and physical activity. Skeletal Muscle Index (SMI), which is derived from limb muscle mass/square of height in kg/m^2^, was used to assess muscle mass (male ≤ 7.0 kg/m^2^, female ≤ 5.7 kg/m^2^); grip strength was used to assess muscle strength (male < 28 kg, female < 18 kg); and 6 m gait speed was used to assess physical performance (speed < 1.0 m/s). The hand grip strength test was performed using a Jamar hydraulic handheld device (Jamar 5030J1; Bolingbrook, USA) to determine the maximum grip strength of the subject's dominant hand in Kilograms (kg). Body composition was measured using an InBody bioelectrical impedance body composition analyzer (InBody S10; Biospace, South Korea) to determine the subjects' muscle mass. The study was approved by the ethics committee in December 2021 (SUDA20211227H03). All subjects were informed of the purpose of the screening and testing tasks. All subjects signed informed consent. This study was designed and reported in accordance with the Strengthening the Reporting of Observational Studies in Epidemiology (STROBE) statement guidelines.

### Inclusion and exclusion criteria

This experiment is a postural balance test. To ensure the safety of the older subjects and minimize the impact of potential factors affecting the postural balance of the older adults on the research results, the inclusion criteria for this study included (1) The older adults aged 60-years and older; (2) Being able to complete sit-to-stand transfers independently and walk on flat ground; (3) Being able to understand the test instructions and perform the test action. Exclusion criteria included (1) Severe diseases such as serious cardiovascular and cerebrovascular disease and advanced cancer; (2) Clearly diagnosed mental illness or cognitive dysfunction, physical disability, injury, or surgery to the extremities within the past three months; (3) Diseases of vestibular function that affect postural balance, such as vestibular neuritis, Meniere's disease, central vestibular lesions, etc. (4) Diseases of visual dysfunction that affect postural balance, such as glaucoma, cataract, macular degeneration, etc. (5) Implantation of artificial joints or metal devices such as cardiac pacemakers.

The screening was based on strict inclusion and exclusion criteria. 37 potential subjects were excluded for severe cardiorespiratory dysfunction, and 24 potential subjects were excluded for failure to perform sit-to-stand transfers and walking independently. In addition, 43 subjects were excluded from the study, including 8 subjects who did not complete the body composition test, 4 subjects who did not complete the grip strength test and 31 subjects who did not complete the other movement tests. Finally, a total of 1026 subjects were enrolled in the study, including 479 males and 547 females.

### Body composition

The measurement of muscle mass was conducted utilizing a segmented multi-frequency method (1 kHz, 5 kHz, 50 kHz, 250 kHz, 500 kHz, and 1 MHz) employing the bioelectrical impedance methods (In-Body S10), an instrument that enables the subject to be measured in a supine or seated position; only the standing position was measured for this study, following the manufacturer's guidelines and the recommendations for clinical use of bioelectrical impedance analysis.^15^ All participants were requested to remove all metal components and to avoid vigorous activity by not eating or drinking for 4 hours prior to testing.

### Postural balance test

#### Static postural balance test

Romberg's test: The subject was instructed to stand naturally with both feet together and drop both upper limbs, stand upright with eyes closed, and observe whether the subject appeared unstable or fell over during the 30 s test. If the time exceeded 30 s, the subject's feet moved, or the body was unstable, the examiner immediately stopped timing and recorded the time, which was accurate to 0.01 s.^16^

Enhanced Romberg's test: The subjects' two feet were held in front of each other, and the tip of the rear foot was tightly attached to the heel of the front foot to maintain a straight line. Subjects adjusted their posture to remain stable and closed their eyes as the examiner began timing.

Single-Leg Stance test with Eyes-Opened (SLS-EO): Subjects were asked to stand on their habitual leg with hands on their hips. The other side of the lower limb could be raised or lowered, but not in contact with the supporting foot.

Single-Leg Stance test with Eyes-Closed (SLS-EC): The test procedure was the same as SLS-EO, but subjects were instructed to close their eyes during the test.^17^

### Dynamic postural balance test

Five Times Sit to Stand Test (FTSST): Prepare an armless chair with a backrest whose sitting position is 45 cm from the floor. Subjects were asked to sit on the chair with arms crossed around their chest during the test. Subjects had to perform five consecutive “sit to stand” movements at the fastest speed without using upper limb strength.^18^

Timed Up & Go test (TUGT): The subject was instructed to sit on a chair, lean the body against the backrest, and place the hands on the armrests. Upon the examiner’s instruction to “Start”, the subject was to stand up from the backrest chair. After standing up firmly, the subject walked forward 3 meters in the daily walking posture to the marked line, turned around, and then returned to the original sitting position to sit down.^19^

Functional Reach Test (FRT): The subject was asked to stand on one side of the wall, feet approximately 10 cm apart, and raise the hands flat. The position of the third metacarpal was measured first. Subjects were asked to bend forward and extend their arms by leaning forward as far as possible without losing physical stability. The forward reach of the third metacarpal was measured, and the number of the originally measured position was subtracted from this result.^17^

Y-Balance Test (YBT): The examiner first measured and noted the length of the subject's left and right legs. The examiner instructed the subject to stand with one leg on the test platform and place their hands on their hips. The subject's other foot stretched forward, posteromedial and posterolateral, as far as possible, and pushed the test plate in all directions slowly and continuously with the tiptoe alternately in all directions to the farthest distance at which balance could be maintained and back to the starting position. The examiner recorded the distance in each direction.^20^ After a rest period of 2‒3 minutes, the subject stood on the other leg and repeated the previous movements.

Prior to the formal administration of the above postural test, subjects were allowed to practice 1‒2 times to familiarize themselves with each test procedure. The entire test was conducted by a team of trained physiotherapists, and to ensure as much standardization as possible, each test was completed by the same team of testers. Subjects are supervised by a protector during the test to prevent falls and accidents.

### Statistical analysis

SPSS 20.0 statistical software (SPSS Science, Chicago, USA) was used for statistical analysis. Sarcopenia prevalence rates were calculated as the number of cases in each age group divided by the total group population, multiplied by 100, and reported as frequencies (n) and percentages ( %). Between-group differences in prevalence were analyzed using the Chi-Square test. The Kolmogorov-Smirnov normality test was performed on all measurements. If they followed the normal distribution, they were described by the mean ± standard deviation. If they did not follow the normal distribution, they were described by the median and interquartile range. Chi-Squared tests were performed for categorical information between the sarcopenia group and the non-sarcopenia group. Measurement data were analyzed by an independent-samples *t*-test. If the normal distribution was not fulfilled, the non-parametric Mann-Whitney *U* nonparametric test was used. Correlations between gender and each variable of the postural balance indicators were analyzed by point-biserial correlation analysis, with values of 0 for men and 1 for women. The age of the population was stratified, with values of 1 for 60‒69 years, 2 for 70‒79 years, and 3 for 80-years and older. Spearman correlation analysis was used to analyze the correlation between the variables of the sarcopenia diagnostic indicator and the variables of the postural balance indicators, and the independent correlation was analyzed by multiple linear regression. Covariate selection follows specific recommendations, including variables that control for exposure or cause of outcome, such as education level, smoking history, alcohol history, etc. The significance level was set at α = 0.05.

## Results

### Sarcopenia screening status

Based on the diagnostic criteria for sarcopenia, subjects were divided into a group with sarcopenia and a group without sarcopenia. The study included 126 patients with sarcopenia and 900 patients without sarcopenia. The prevalence of sarcopenia was 12.28 %. The basic information of the subjects is shown in Table 1. Among the patients with sarcopenia, 55 were males with a prevalence of 11.48 % and 71 were females with a prevalence of 12.98 %. The prevalence of sarcopenia in males and females was statistically significant (*t* = 6.539, *p* < 0.01).

**Table 1:** Baseline of subjects.

Variables	Sarcopenia (*n* = 126)	Non-sarcopenia (*n* = 900)
Age, years	72.47 ± 4.81	71.23 ± 5.60
Gender, female n ( %)	71 (56.35 %)	476 (52.89 %)
Weight, kg	57.56 ± 8.19	60.82 ± 9.59
Height, m	1.60 ± 0.08	1.62 ± 0.08
BMI, kg/m^2^	22.48 ± 2.42	24.41 ± 3.90
SMI, kg/m^2^	6.07 ± 0.35	7.64 ± 0.27
Educational level, > 9-years n ( %)	70 (55.56 %)	516 (57.33 %)
Ever/current smoke, yes n ( %)	21 (16.67 %)	9.89 (9.89 %)
Ever/current alcohol, yes n ( %)	17 (13.49 %)	93 (10.33 %)
Comorbidities (including high blood pressure, high blood cholesterol, and hyperglycemia) n ( %)	72(57.14 %)	372(41.44 %)

**Fig. 1:**
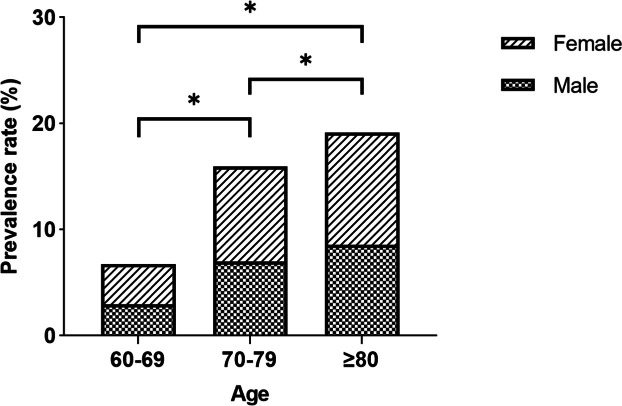
Prevalence of sarcopenia, *n* = 126 (*p < 0.05).

Based on age stratification, the age groups of sarcopenia in this study were categorized as 60‒69 years, 70‒79 years, 80-years and above. The prevalence of sarcopenia in each stage was 6.71 % (*n* = 22), 15.88 % (*n* = 74), and 18.99 % (*n* = 30), respectively. There was a significant difference in the prevalence of sarcopenia in all age groups, *p* < 0.05 (Fig. 1).

### Correlation analysis of static postural balance in sarcopenia

According to the screening results of sarcopenia, based on the characteristics of sarcopenia, gender, age factors and diagnostic factors of sarcopenia were included to further analyze the correlation of the sarcopenia population. In Table 2, the correlation analysis of static postural balance in the sarcopenia group showed that there was no significant correlation between gender, age stratification, hand grip, 6 m gait speed, SMI, and Romberg's test (*p* > 0.05); there was a weak correlation between age stratification, hand grip, and Enhanced Romberg's test (*r* = −0.228, *p* = 0.029; *r* = 0.277, *p* = 0.008); age stratification was weakly correlated with SLS-EO (*r* = −0.221, *p* = 0.034), and hand grip and 6 m gait speed were moderately and weakly correlated with SLS-EC, respectively (*r* = 0.317, *p* = 0.002; *r* = 0.268, *p* = 0.01). In the single-leg stance test with eyes closed, hand grip and SMI were strongly correlated with SLS-EC (*r* = 0.267, *p* = 0.01), and 6 m gait speed was weakly correlated with SLS-EC (*r* = 0.267, *p* = 0.01). There was no significant correlation between age and SLS-EC (*p* > 0.05).

**Table 2:** Correlation analysis of static postural balance in sarcopenia (*n* = 126).

Variable	Romberg's test		Enhanced Romberg's test		SLS-EO		SLS-EC	
	*r*	p	*r*	p	*r*	p	*r*	p
Gender	0.110	0.296	0.021	0.842	−0.018	0.867	−0.470	0.050
Age stratification	−0.256	0.140	−0.228	**0.029**	−0.221	**0.034**	−0.073	0.487
Hand Grip	0.032	0.846	0.277	**0.008**	0.317	**0.002**	0.681	**<0.001**
6 m gait speed	0.021	0.900	0.135	0.199	0.268	**0.010**	0.267	**0.010**
SMI	−0.091	0.387	0.111	0.293	0.076	0.471	0.550	**<0.001**

Note: p-values in bold indicate significant differences.

**Fig. 2:**
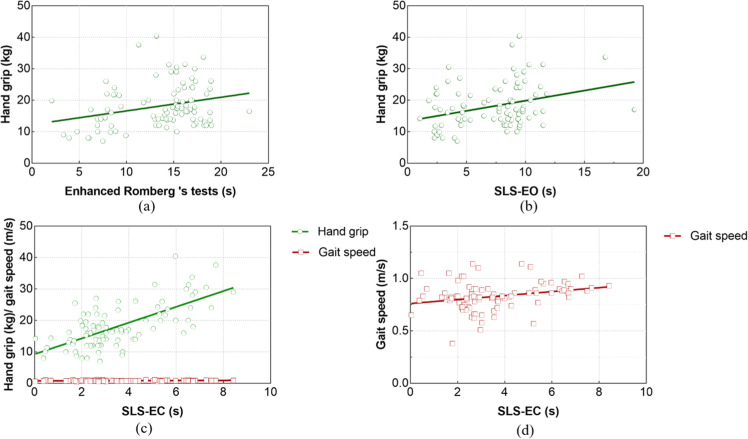
Regression analysis of static postural balance in sarcopenia (*n* = 126). (d refer to the local amplification of the gait speed variable in c).

After Romberg's test was included as the dependent variable in the regression analysis, hand grip was identified as a predictor variable (*t* = 2.142, *p* = 0.03). Hand grip had a significant and positive effect on the duration of the Enhanced Romberg's test in people with sarcopenia. For each unit (kg) increased in hand grip, the time of Enhanced Romberg's test increased by 0.144 (95 % CI: 0.010∼0.278) units (s), as shown in Fig. 2.

After entering the regression analysis with SLS-EO as the dependent variable, the screened predictor variable was hand grip (*t* = 2.363, *p* = 0.02). Hand grip significantly and positively influenced the time of SLS-EC in subjects with sarcopenia. For each unit (kg) increased in hand grip, the time of SLS-EC increased by 0.124 (95 % CI: 0.020∼0.229) units.

After SLS-EC was included as the dependent variable in the regression analysis, the predictor variables selected were hand grip (*t* = 4.359, *p* < 0.001) and 6 m gait speed (*t* = 1.173, *p* = 0.044). Hand grip and 6 m gait speed significantly and positively influenced the time of SLS-EC. For each unit (kg) increase in the hand grip, the time of SLS-EC increased by 0.153 (95 % CI 0.085∼0.221) units (s). For each unit (m/s) increase in 6 m gait speed, the test time of SLS-EC increased by 1.394 (95 %CI: −0.969∼3.758) units.

The specific regression coefficients and effect tests for the above models are shown in Table 3.

**Table 3:** Regression coefficient and effect test (*n* = 126).

	Regression coefficient B	*t*	p	95 % CI	Collinearity	
					Tolerance	VIF
**Gender**						
Model: FRT	0.464	0.353	0.725	−2.145, 3.072	0.176	5.681
**Age stratification**						
Model: Enhanced Romberg's test	−1.093	−1.562	0.122	−2.484, 0.298	0.906	1.103
Model: SLS-EO	−0.188	−0.346	0.73	−1.264, 0.889	0.875	1.142
Model: FTSST	1.27	1.976	0.051	−0.007, 2.547	0.934	1.070
Model: TUGT	1.816	2.693	0.008	0.476, 3.156	0.875	1.142
Model: FRT	−0.612	−1.373	0.173	−1.498, 0.274	0.855	1.170
Model: YBT-Left score	−11.016	−2.897	0.007	−18.818, −3.214	0.803	1.245
Model: YBT-Right score	−9.21	−2.484	0.020	−16.818, −1.602	0.803	1.245
**Hand grip**						
Model: Enhanced Romberg's test	0.144	2.142	0.035	0.010, 0.278	0.906	1.103
Model: SLS-EO	0.124	2.363	0.020	0.020, 0.229	0.860	1.163
Model: SLS-EC	0.153	4.445	<0.001	0.085, 0.221	0.372	2.687
Model: TUGT	−0.108	−1.645	0.013	−0.247, −0.022	0.860	1.163
Model: FRT	0.196	2.901	0.005	0.062, 0.330	0.343	2.916
**6m gait speed**						
Model: SLS-EO	4.803	1.762	0.081	−0.613, 10.218	0.886	1.129
Model: SLS-EC	1.394	1.173	0.044	−0.969, 3.758	0.864	1.158
Model: FTSST	−11.669	−3.59	0.001	−18.129, −5.210	0.934	1.070
Model: TUGT	−14.439	−4.258	<0.001	−21.177, −7.701	0.886	1.129
Model: FRT	2.934	1.29	0.021	1.588, 7.456	0.840	1.191
Model: YBT-Left score	23.267	1.763	0.008	3.813, 50.347	0.803	1.245
Model: YBT-Right score	28.033	2.178	0.038	1.626, 54.440	0.803	1.245
**SMI**						
Model: SLS-EC	0.364	0.975	0.332	−0.377, 1.105	0.404	2.476
Model: FRT	0.155	0.122	0.904	−2.380, 2.690	0.123	8.138

Note: p-values in bold indicate significant differences.

### Correlation analysis of dynamic postural balance in sarcopenia

According to the correlation analysis of dynamic postural balance in the sarcopenia group in Table 4, there was no significant correlation between gender, age stratification, hand grip, 6 m gait speed, SMI, and YBT-Bilateral differences (*p* > 0.05); Age and 6 m gait speed were moderately correlated with FTSST (*r* = 0.309, *p* = 0.003; *r* = −0.403, *p* < 0.001); There was a moderate correlation between age stratification and TUGT (*r* = 0.449, *p* < 0.001), while hand grip and 6 m gait speed were weakly and moderately correlated with TUGT (*r* = −0.341, *p* = 0.001; *r* = −0.498, *p* < 0.001); age stratification was strongly correlated with YBT-Left score and YBT-Right score (*r* = −0.629, *p* < 0.001; *r* = −0.618, *p* < 0.001); 6 m gait speed was moderately and strongly correlated with YBT-Left score and YBT-Right score, respectively (*r* = 0.497, *p* = 0.005; *r* = 0.537, *p* = 0.002). There was a weak correlation between age stratification and FRT (*r* = −0.262, *p* = 0.012), a moderate correlation between SMI and FRT (*r* = 0.333, *p* = 0.001), and a strong correlation between hand grip and FRT (*r* = 0.503, *p* < 0.001). Gender and 6 m gait speed were weakly correlated with FRT (*r* = −0.269, *p* = 0.009; *r* = 0.290, *p* = 0.005).

**Table 4:** Correlation analysis of dynamic postural balance in sarcopenia (*n* = 126).

Variable	FTSST		TUGT		FRT		YBT-Left score		YBT-Right score		YBT-Bilateral differences	
	*r*	p	*r*	p	*r*	p	*r*	p	*r*	p	*r*	p
Gender	−0.005	0.961	0.039	0.710	−0.269	**0.009**	0.013	0.944	−0.003	0.989	0.120	0.255
Age stratification	0.309	**0.003**	0.449	**<0.001**	−0.262	**0.012**	−0.629	**<0.001**	−0.618	**<0.001**	0.138	0.190
Hand Grip	−0.160	0.128	−0.341	**0.001**	0.503	**< 0.001**	0.219	0.245	0.244	0.194	−0.112	0.290
6 m gait speed	−0.403	**<0.001**	−0.498	**<0.001**	0.290	**0.005**	0.497	**0.005**	0.537	**0.002**	−0.179	0.087
SMI	−0.034	0.747	−0.101	0.337	0.333	**0.001**	0.233	0.216	0.238	0.255	−0.080	0.446

Note: p-values in bold indicate significant differences.

**Fig. 3:**
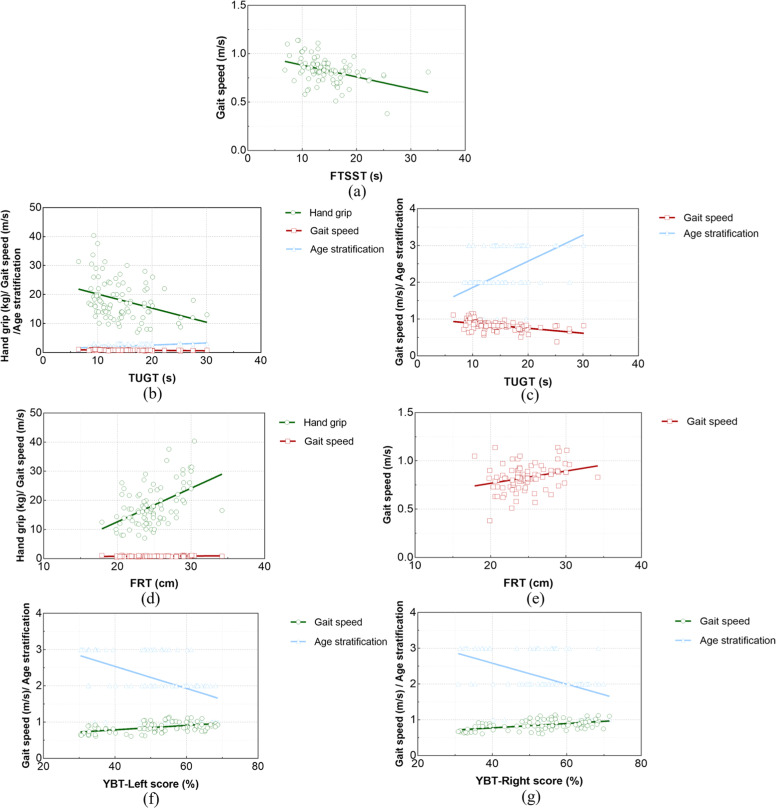
Regression analysis of dynamic postural balance in sarcopenia (*n* = 126). (c refers to the local amplification of the gait speed and age stratification variables in b; e refers to the local amplification of the gait speed variable in d).

After FTSST was included as a dependent variable in the regression analysis, the screened predictor variable was 6 m gait speed (*t* = −3.590, *p* = 0.001). 6 m gait speed significantly and negatively affected the time of FTSST in individuals with sarcopenia. For each unit (m/s) increase in 6 m gait speed, the time of FTSST increased by 11.669 (95 % CI: −18.129∼−5.210), as shown in Fig. 3.

After entering the regression analysis with TUGT as the dependent variable, the screened predictor variables were age stratification (*t* = 2.693, *p* = 0.008), hand grip (*t* = −1.645, *p* = 0.013), and 6 m gait speed (*t* = −4.678, *p* < 0.001). Age significantly and positively affected the time of TUGT in subjects with sarcopenia. For each unit (stratification+1) increased in age, the time of TUGT increased by 1.186 (95 % CI: 0.476∼3.156) units. Both hand and 6 m gait speed significantly negatively affected the time of TUGT in people with sarcopenia. For each unit (kg) increased in the hand grip, the time of TUGT decreased by 0.108 (95 % CI: −0.237∼−0.022) units (s). The time of TUGT decreased by 14.439 (95 % CI: −21.177∼−7.701) units (s) per unit (m/s) at 6 m gait speed.

After FRT was used as the dependent variable in the regression analysis, the screened predictive variables were hand grip (*t* = 2.901, *p* = 0.005) and 6 m gait speed (*t* = 1.290, *p* = 0.021). Both hand grip and 6 m gait speed significantly positively affected the distance of FRT in people with sarcopenia. For each unit (kg) increased in hand grip, the distance of FRT increased by 0.196 (95 % CI: 0.062∼0.330) units (cm). The distance of FRT increased by 2.934 (95 % CI: 1.588∼7.456) units (cm) per unit (m/s) at 6 m gait speed.

After entering the regression analysis with YBT-Left score as the dependent variable, the screened predictive variables were age stratification (*t* = −2.897, *p* = 0.007) and 6 m gait speed (*t* = 1.763, *p* = 0.008). Age had a significant negative effect on the YBT-Left score in subjects with sarcopenia. For each unit (stratification+1) increased in age, YBT-Left score decreased by 11.016 (95 % CI: −18.818∼−3.214) units ( %). 6 m gait speed significantly positively affected the YBT-Left score in people with sarcopenia. For each unit (m/s) increase in 6 m gait speed, YBT-Left score increased by 23.267 (95 % CI: 3.813∼50.347) units ( %).

After YBT-Right score was used as the dependent variable in the regression analysis, the screened predictive variables were age stratification (*t* = −2.484, *p* = 0.020) and 6 m gait speed (*t* = 2.178, *p* = 0.038). YBT-Right score decreased by 9.210 (95 % CI: −16.818∼−1.602) units ( %) for each unit (stratification+1) increased in age stratification. 6 m gait speed significantly positively affected the YBT-Right score for people with sarcopenia. For each unit (m/s) increase in 6 m gait speed, the YBT-Left score increased by 28.033 (95 % CI: 1.626∼54.440) units ( %).

Specific regression coefficients and effect tests of the above models are shown in Table 3.

## Discussion

### Analysis of screening results for sarcopenia

In this study, the prevalence of sarcopenia was investigated according to the diagnostic criteria for sarcopenia proposed by the AWGS in 2019. The results showed that the prevalence of sarcopenia differed between men and women, and the prevalence of sarcopenia was slightly higher in women than in men. A study on the prevalence of sarcopenia in Taiwan found 6.5 % in men and 8.2 % in women, with a higher prevalence in men than in women.^21^ Another study of the prevalence of sarcopenia in older adults over 70 years of age in the community of the UK showed that the prevalence of sarcopenia in men and women was 7.8 % and 7.9 %, respectively.^22^ The prevalence of sarcopenia in men and women varied between studies, taking into account factors such as the use of diagnostic criteria for sarcopenia, detection methods, instruments, and different population characteristics in the study area. For this reason, in this part of the study, the sarcopenia population was divided by gender before statistical comparisons. Meanwhile, this study stratified the sarcopenia population based on an age interval of ten years. The results showed a significant increase in the prevalence of sarcopenia with increasing age, similar to previous studies. A stratified survey of the prevalence of sarcopenia in Taiwan, conducted by Lin et al.^23^ showed that the prevalence of sarcopenia was only 7.4 % at the age of 65‒74 years, while it increased to over 17.5 % at the age of 75 years. It can be seen that sarcopenia has some prevalence in older adults with some gender differences. Aging is one of the major factors contributing to the increased prevalence of sarcopenia.

### Relationship between skeletal muscle aging and postural balance

Age-related muscle atrophy is an important sign in the diagnosis of sarcopenia. A quantitative study of skeletal muscle in older adults has shown that the rate of skeletal muscle loss may exceed 0.65 % per year in older adults over 75-years of age, and the rate of muscle loss will continue to increase in older adults who lack exercise habits and adequate nutrition.^24^ Skeletal muscle aging is often accompanied by some changes in the structure and function of muscles, gradually limiting the body's ability to participate in daily activities.^2^ Studies have shown that even in healthy older people, age-related functional decline of the sensory, motor, and neuromuscular systems has a greater negative impact on the performance of human postural balance^25^. Postural balance is the ability of the body to automatically adjust and maintain postural stability when sitting and standing statically, moving spontaneously, and being disturbed by external forces. Maintaining human postural balance requires the coordination of visual, vestibular, proprioceptive, and muscular control. Compared with physical qualities such as agility, flexibility, and coordination, older adults have a more pronounced decline in balance function.^26^^,^^27^ A Stable balance function is the basic guarantee of older adults’ ability to perform their daily activities. Balance dysfunction inevitably affects the daily life of older adults and reduces their quality of life.^28^

In this study, gender, age, and three diagnostic indicators of muscle strength, gait speed, and SMI were analyzed in correlation with static and dynamic postural balance tests, respectively, in the sarcopenia population. The results of the correlation analysis of static postural balance showed that there was no correlation between gender and static postural balance in sarcopenia. Age was negatively correlated with static postural balance, whereas hand grip, gait speed, and SMI were positively correlated with static postural balance, which was basically consistent with the results of previous studies.^29^ In the aging process, the decline in muscle mass and functional level of skeletal muscle is probably the most significant of all changes, and the progressive decline in muscle mass with age gradually affects the maintenance of skeletal muscle strength.^2^ Studies have revealed that the accelerated loss of muscle mass and function is closely associated with body balance function and the occurrence of fall-related adverse events.^30^ Furthermore, other studies have shown that a decline in muscle strength will reduce the ability of older adults to maintain body posture and stabilize movements, leading to balance disorders.^31^ Skeletal muscle atrophy due to aging may have a greater impact on dynamic postural balance than on static postural balance. The results of this study demonstrated that the diagnostic indicators of sarcopenia correlated more strongly with FTSST, TUGT, FRT, YBT-Left score, and YBT-Right score. Among them, gait speed correlated moderately with all of the dynamic postural balance tests described above, suggesting that maintaining dynamic posture in people with sarcopenia may depend more on the stability of muscle activity function. YBT is a comprehensive functional test that reflects the dynamic postural stability of the subjects’ lower extremities and left-right balance dysfunction, with a reliability score of 0.95 in the older adults.^20^ At the same time, an unhealthy sedentary lifestyle is an important influencing factor in causing sarcopenia. Atrophy and loss of skeletal muscle mass in the lower extremities are often associated with decreased neuromuscular control function.^32^ However, performing YBT requires good lower limb strength and proprioception in subjects. People with sarcopenia tend to have decreased proprioceptive function due to muscle fatigue, which is due to the decrease in the discharge rate of α-motor neurons and decreased ionic number Ca^2+^ at the neuromuscular junction.^33^

One of the primary functional goals of postural control is to achieve postural balance. Postural balance relies on the self-triggering of the body's postural stability and the integration of sensory and motor strategies to stabilize body mass in response to external perturbations. Achieving this multi-system coordination and adjustment of movement strategies requires good neuromuscular performance.^34^^,^^35^ Previous studies have shown that age-related declines in neuromuscular function manifest in a variety of ways. Atrophy of skeletal muscle fibers, morphological changes and characteristic loss of motor neurons, as well as instability of neuromuscular connections, may result in different isoforms of myosin heavy chain expressed in muscle fibers of older adults, affecting muscle performance and complicating motor control in older adults. Sophisticated and varied requirements for motor control may better reflect age-related changes in postural balance.^36^^,^^37^ The results of multiple linear regression analysis in this study showed that an increase in age and a decrease in hand grip and gait speed were the main factors leading to a decrease in static and dynamic postural balance in people with sarcopenia. For the static postural balance test, Enhanced Romberg's test, SLS-EO, and hand grip (muscle strength) are the most critical factors affecting their test results. For the dynamic postural balance test, any change in TUGT, FRT, gait speed (somatic activity function), age, and muscle strength can have a considerable impact on dynamic postural stability. This discrepancy might be indicative of the hierarchical vulnerability of neural vs. muscular systems. While compensatory cognitive strategies can partially maintain static stability, dynamic perturbations exceed cortical processing speeds, thus unmasking the neuromuscular deficits associated with sarcopenia.^38^ Notably, this accelerated sarcopenic progression disproportionately affects type II muscle fibres, which are critical for rapid force generation during dynamic balance adjustments.^39^ Therefore, the older the age, the lower the muscle strength, the lower the somatic activity function, and the poorer the dynamic control ability, which further supports the theory of postural balance. Simultaneously, the findings may reveal that the static postural balance of people with sarcopenia can be improved by strengthening muscles, while the improvement of physical function and muscle strength should be emphasized to promote the dynamic postural balance of people with sarcopenia. Moreover, due to the objectivity of age factors, the progressive decline of dynamic postural balance of older people is inevitable.

In terms of gender, the correlation analysis in this study showed that other static and dynamic postural balance indices were not significantly correlated with gender, except FRT. Previous studies have shown that body height has a significant effect on the distance of FRT^40^, and the average height of men is generally higher than that of women, which is considered to be the reason for the correlation analysis between FRT and sarcopenia. Meanwhile, when gender was included in the multiple linear regression analysis in this study, the low regression coefficient and non-significant difference indicated that gender may not be an important factor affecting the dynamic postural balance of people with sarcopenia. Moreover, due to the high collinearity of the SMI (tolerance < 0.5), this parameter was ultimately not included in the regression equation of static and dynamic postural balance.

### Limitation

There are several limitations to this study. First of all, although this study attempted to control the factors that may affect the postural balance of the sarcopenia population, such as age, gender, and muscle mass, the sample size was not large enough to conduct a more detailed stratified analysis of the sample, and other confounding factors that may affect the regression analysis were not fully considered. Future studies will further increase the sample size of sarcopenia to improve the accuracy of the results. Moreover, with subject recruitment in only one city and fewer subjects over the age of 80, the analyses obtained in this study may not adequately reflect the postural balance characteristics of all Asian sarcopenia populations.

## Conclusion

The prevalence of sarcopenia is higher in women than in men, and it increases significantly with age. In terms of postural balance, age-induced skeletal muscle aging may have a more pronounced effect on dynamic postural balance. Age is an inevitable factor in the decline of dynamic postural balance in people with sarcopenia. Currently, there is no direct evidence that gender may affect postural balance in people with sarcopenia. The static postural balance of people with sarcopenia can be improved by targeted resistance training focusing on muscle strength, whereas enhancing dynamic postural balance necessitates integrated protocols combining muscle strengthening with task-specific functional exercises.

## Abbreviations

AWGS, Asian Working Group for Sarcopenia; FTSST, Five Times Sit to Stand Test; SLS, Single Leg Standing Test; SLS-EO, Single-Leg Stance test with Eyes-Opened; SLS-EC, Single-Leg Stance test with Eyes-Closed; SMI, Skeletal Muscle Index; TUGT, Timed Up & Go Test; YBT, Y-Balance Test.

## Approval for human experiments

Written informed consent was obtained from the patient for publication of this study.

## Date availability statement

The data that support the findings of this study are available from the corresponding author upon reasonable request.

## Authors’ contributions

Cenyi Wang, Aming Lu: Conceptualization, Methodology, Software. Cenyi Wang, Xinrong Jiao and Jiling Liang: Data curation, Writing-original draft preparation. Aming Lu: Supervision. Cenyi Wang, Xinrong Jiao: Writing-reviewing and editing.

## Funding

This research was supported by the Postdoctoral Fellowship Program of CPSF (GZC20231899), Major Sports Research Project of Jiangsu Sports Bureau (ST242106), and the Youth Foundation Project of Humanities and Social Sciences Research of the Ministry of Education (18YJC890001). The funder had and will not have a role in any of the aspects of the study design, data collection analysis, publication, or development of the manuscript.

## Declaration of competing interest

The authors declare that there are no conflicts of interest regarding the publication of this paper.
